# NADPH Oxidase NOX4 Mediates Stellate Cell Activation and Hepatocyte Cell Death during Liver Fibrosis Development

**DOI:** 10.1371/journal.pone.0045285

**Published:** 2012-09-26

**Authors:** Patricia Sancho, Jèssica Mainez, Eva Crosas-Molist, César Roncero, Conrado M. Fernández-Rodriguez, Fernando Pinedo, Heidemarie Huber, Robert Eferl, Wolfgang Mikulits, Isabel Fabregat

**Affiliations:** 1 Biological Clues of the Invasive and Metastatic Phenotype Group, Bellvitge Biomedical Research Institute (IDIBELL), L'Hospitalet, Barcelona, Spain; 2 Departamento de Bioquímica y Biología Molecular II, Facultad de Farmacia, Universidad Complutense and Instituto de Investigación Sanitaria del Hospital Clínico San Carlos (IdISSC), Madrid, Spain; 3 Service of Gastroenterology, Hospital Universitario Fundación Alcorcón and Universidad Rey Juan Carlos, Alcorcón, Madrid, Spain; 4 Unit of Pathology, Hospital Universitario Fundación Alcorcón, Alcorcón, Madrid, Spain; 5 Department of Medicine I, Division: Institute of Cancer Research, Medical University of Vienna, Vienna, Austria; 6 Ludwig Boltzmann Institute for Cancer Research (LBI-CR), Vienna, Austria; 7 Department of Physiological Sciences II, University of Barcelona, Barcelona, Spain; Leiden University Medical Center, The Netherlands

## Abstract

A role for the NADPH oxidases NOX1 and NOX2 in liver fibrosis has been proposed, but the implication of NOX4 is poorly understood yet. The aim of this work was to study the functional role of NOX4 in different cell populations implicated in liver fibrosis: hepatic stellate cells (HSC), myofibroblats (MFBs) and hepatocytes. Two different mice models that develop spontaneous fibrosis (Mdr2^−/−^/p19^ARF−/−^, Stat3^Δhc^/Mdr2^−/−^) and a model of experimental induced fibrosis (CCl_4_) were used. In addition, gene expression in biopsies from chronic hepatitis C virus (HCV) patients or non-fibrotic liver samples was analyzed. Results have indicated that NOX4 expression was increased in the livers of all animal models, concomitantly with fibrosis development and TGF-β pathway activation. *In vitro* TGF-β-treated HSC increased NOX4 expression correlating with transdifferentiation to MFBs. Knockdown experiments revealed that NOX4 downstream TGF-β is necessary for HSC activation as well as for the maintenance of the MFB phenotype. NOX4 was not necessary for TGF-β-induced epithelial-mesenchymal transition (EMT), but was required for TGF-β-induced apoptosis in hepatocytes. Finally, NOX4 expression was elevated in patients with hepatitis C virus (HCV)-derived fibrosis, increasing along the fibrosis degree. In summary, fibrosis progression both *in vitro* and *in vivo* (animal models and patients) is accompanied by increased NOX4 expression, which mediates acquisition and maintenance of the MFB phenotype, as well as TGF-β-induced death of hepatocytes.

## Introduction

Liver fibrosis is the final consequence of many chronic liver injuries [1]. Hepatic stellate cells (HSCs) are activated to myofibroblasts (MFBs), which are mainly responsible for collagen deposition during hepatic fibrogenesis. Once injured, hepatocytes undergo apoptosis. The transforming growth factor-beta (TGF-β), whose levels increase during the development of liver fibrosis, could be involved in both processes [2]. Thus, TGF-β inhibits growth and induces apoptosis of hepatocytes and also contributes to the activation of HSCs [3], [4].

The generation of reactive oxygen species (ROS) plays relevant roles in hepatic fibrosis and recent works point to NADPH oxidases (NOX) as a key source of ROS in the fibrotic liver [5]. Two NOX isoforms, NOX1 and NOX2, mediate pro-fibrogenic effects in endogenous liver cells [6], [7], [8]. However, less is known about the possible role in liver fibrosis of another isoform, NOX4, which is highly expressed in hepatocytes and HSCs [8]. We previously reported that NOX4 mediates TGF-β-induced apoptosis in hepatocytes in primary culture [9] and causes ROS production upon the *in vitro* transdifferentiation of activated HSCs to MFBs [3]. In other fibrotic models, NOX4 accounts for ROS-induced fibroblast and mesangial cell activation, playing an essential role in TGF-β1-mediated fibroblast differentiation into a profibrotic myofibroblast phenotype and matrix production [10]. Indeed, TGF-β induces NOX4 expression in lung mesenchymal cells, which mediates MFB activation and fibrogenic responses to lung injury [11]. In this same line of evidence, ROS signaling by NOX4 is required for TGF-β-induced differentiation of fibroblasts into MFB in heart [12], kidney [13] and diseased prostatic stroma [14].

The aim of this work was to analyze whether NOX4 expression is modulated in experimental animal models of liver fibrosis and during the development of human liver fibrogenesis. We demonstrate that NOX4 expression increases in parallel to liver fibrotic processes and may be required for TGF-β-induced activation of HSC and for the maintenance of the MFB phenotype. In hepatocytes, NOX4 causes cell death but does not mediate epithelial-mesenchymal transition (EMT). These results open new perspectives for the involvement of NOXes in liver fibrosis and for the potential development of new therapeutic targeted tools.

## Materials and Methods

### Ethics statement

Mice were housed in accordance with European laws and with the general regulations specified by the Good Scientific Practices Guidelines of the Medical University of Vienna. From Spain, the approval for all the experiments related to the study of liver fibrosis in experimental animal models was applied to the General Direction of Environment and Biodiversity, Government of Catalonia, and approved with the number #4589, 2011 (document enclosed). Human tissues were collected with the required approvals from the Institutional Review Board (Comité Ético de Investigación Clínica del Hospital Universitario Fundación Alcorcón) and patient's written consent conformed to the ethical guidelines of the 1975 Declaration of Helsinki (both documents are enclosed).

### Reagents and antibodies

TGF-β was from Merck (Darmstadt, Germany). Fetal bovine serum was from Sera Laboratories International (Cinder Hill, UK). Glutathione-ethyl-ester (GEE), Diphenyleneiodonium chloride (DPI) and Butylated hydroxyanisole (BHA) were from Sigma (St Louis, USA). The caspase-3 substrate Ac-DEVD-AMC was from Pharmingen (San Diego, CA, USA). Antibodies: mouse anti-β-actin (clone AC-15, Sigma), rabbit anti-cleaved caspase-3 (Asp-175) from Cell Signaling Technology (Danvers, MA, USA), anti-F4/80 (Abcam, Cambridge, UK), mouse anti-E-cadherin (BD Pharmingen, NJ, USA), rabbit anti-ki67 (Abcam), mouse anti-NOX2 (Santa Cruz Biotechnology, CA, USA), anti-NOX4 raised by Sigma-Genosys against a peptide corresponding to the C-terminal loop region (aminoacids 499–511), mouse anti-α-SMA (Sigma, St Louis, USA), rabbit anti-phospho-Smad2 (Ser465/467) and rabbit anti-phospho-Smad3 (Ser423/425) from Cell Signaling Technology, goat anti-Smad2/3, anti-Smad7 and anti-TGF-β from Santa Cruz Biotechnology and mouse anti-vimentin (Sigma, St Louis, USA).

### Mice

Three animal experimental models of liver fibrosis were used for this study: two genetically modified mice and one drug-induced model. Mdr2^−/−^/p19^ARF−/−^ double null mice [15] displayed a fibrotic phenotype comparable to Mdr2^−/−^ mice, widely used as a model for experimental liver fibrosis [16], [17], characterized by severe hepatic injury and large periductal accumulation of MFBs, but showed the additional advantage of allowing the isolation of immortal cells for *in vitro* experiments [15]. Stat3^Δhc^/Mdr2^−/−^ mice show Stat3 conditional inactivation specifically in hepatocytes and cholangiocytes in a Mdr2^−/−^ background [18], which strongly aggravates liver injury and fibrosis. Animals were sacrificed to evaluate liver histology at 2 and 7 weeks of age. C.B-17/IcrHsd-Prkcd^scid^ (SCID) mice have been used as control. For CCl_4_ experiments, CCl_4_ (Sigma, St Louis, USA) was diluted 1∶4 in ultrapure Olive Oil (Sigma, St Louis, USA) and injected intraperitoneally at a concentration of 1.5 mg per gram of body weight into 3-month old C57BL/6J mice. The treatment was performed two times a week for 6 weeks to induce hepatic fibrosis. Mice were sacrificed 3 days after the last injection and liver samples were collected and processed for immunohistochemical analysis.

### Cell models and cell culture conditions

No commercial cell lines have been used in this study. Mice stellate cells, myofibroblasts and hepatocytes were obtained in our or colleagues' laboratories and used in previous published works [14], [18], [19]: 1) By employing p19^ARF^ deficiency, we established a non-transformed murine HSC model to investigate their plasticity and the dynamics of HSC activation [19]. The immortal cell line, referred to as M1-4HSC, showed stellate cell characteristics including the expression of desmin, glial fibrillary acidic protein, alpha-smooth muscle actin and pro-collagen I [19]. Treatment of these non-tumorigenic M1-4HSC with pro-fibrogenic TGF-β1 provoked a morphological transition to a myofibroblastoid cell type which was accompanied by enhanced cellular turnover and impaired migration [19]. These cells have been used in this study to analyze the role of NOX4 in the *in vitro* activation of HSC to MFB; 2) *In vivo*-activated MFBs derived from physiologically inflamed livers of Mdr2/p19^ARF^ double-null mice were obtained as previously described [15] and used in this study in *in vitro* experiments to analyze the role of NOX4 in maintaining the myofibroblast phenotype; 3) Finally, hepatocytes from wild-type mice were isolated and immortalized with a puromycin-resistance retroviral vector pBabe encoding Simian virus 40 large T antigen (LTAg), as described [20] and were generously provided by Dr. AM Valverde (Madrid, Spain). These cells were used in this study to analyze the potential role of NOX4 in the TGF-β-induced effects related to fibrosis development, i.e., epithelial-mesenchymal transitions and apoptosis. For cell culture, cells were grown in DMEM (Lonza, Basel, Switzerland) supplemented with 10% FBS and maintained in a humidified atmosphere of 37°C, 5% CO2.

### Human samples

Biopsies from 28 chronic hepatitis C virus (HCV) patients showing different degrees of fibrosis or control liver samples extracted in surgeries from colorectal cancer patients with hepatic metastasis were collected in the Hospital Universitario de Alcorcón (Madrid, Spain). The control group was formed by 10 patients in whom liver biopsy was performed by altered function test. Liver biopsy examination revealed normal histology or minimal change.

### Immunohistochemistry and immunocytochemistry studies

For immunohistochemistry, sections of 4 μm-thick paraffin-embedded livers were stained with hematoxylin and eosine (H&E) or Trichrome (Sigma) for collagen staining using standard procedures. The immunostaining was performed by incubating primary antibodies (diluted from 1∶50 to 1∶100) overnight at 4°C and by visualization with the Vectastain ABC kit (Vector Laboratories, Burlingame, CA). Immunocytochemistry studies were performed as described previously [21]. Representative images were taken with a Spot 4.3 digital camera and edited in Adobe Photoshop. Cells were visualized in an Olympus BX-60 with the appropriate filters.

### Analysis of cell number

Cell number was analyzed after crystal violet staining [4].

### Total ROS production

Intracellular ROS content was measured by staining with the fluorescent probe H_2_DCF-DA as described previously [22].

### Analysis of caspase-3 activity

Caspase-3 activity was analyzed fluorimetrically upon incubation of 20 μg of cell lysates with 6.6 μg/mL Ac-DEVD-AMC for 2 hours at 37°C [22]. Results are calculated as units of caspase-3 activity per microgram of protein per hour.

### Analysis of gene expression

RNeasy Mini Kit (Qiagen, Valencia, CA, USA) was used for total RNA isolation. Reverse transcription (RT) was carried out using the High Capacity Reverse Transcriptase kit (Applied Biosystems, Foster City, CA, USA), and 500 ng of total RNA from each sample for complementary DNA synthesis. PCR products in semiquantitative reactions were obtained after 30–35 cycles of amplification at annealing temperatures of 57–62°C, and analyzed by 1.5% agarose gel electrophoresis. Expression of *18S* was analyzed as a loading control, as indicated. The –RT channel contained RNA that had not been treated with the RT mixture.

For Real-Time quantitative PCR, expression levels were determined in duplicate in an ABIPrism7700 System, using the Sybr® Green PCR Master Mix (Applied Biosystems).

All the primers used for both semiquantitative PCR or Real-Time quantitative PCR reactions are listed in Suppl. Table 1 and 2, respectively.

### Western blot analysis

Total protein extracts and Western Blot procedure were carried out as previously described [22], [23]. Antibodies were used at 1∶1000, except β-actin (1∶3000). Protein concentration was measured with the BCA^TM^ Protein Assay kit (Pierce, Rockford, USA).

### Knock-down assays

Cells at 70% confluence were transiently transfected with 50 nM siRNA during 8 hours using TransIT-siQuest following manufacturer's instructions (Mirus, Madison, USA). Oligos were obtained from Sigma-Genosys (Suffolk, UK). The oligo sequences were as follows: unsilencing: GUAAGACACGACUUAUCGC; mouse NOX4: CAAGAAGAUUGUUGGAUAA. The unsilencing siRNA used was selected from previous works [22]. Specific oligos with maximal knock-down efficiency were selected among three different sequences for each gene.

### Statistics

All data represented at least three experiments and expressed as the mean ± SEM. Differences between groups were compared using either Student's t test or one-way ANOVA associated with the Dunnett's test. Statistical significance was assumed when p<0.05.

## Results

### Activation of the TGF-β/NOX4 pathway in fibrosis development

**Figure 1:**
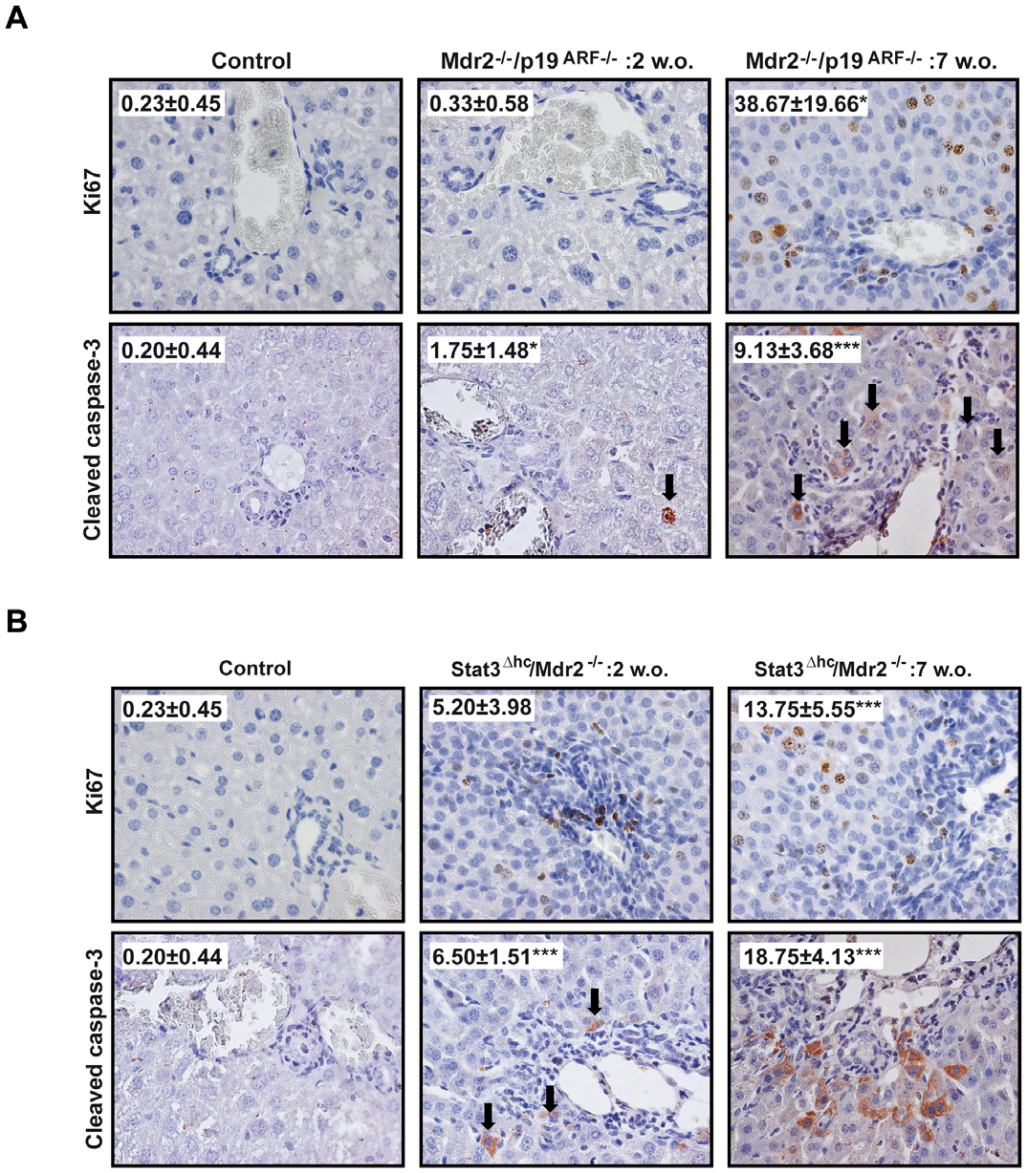
Fibrosis development in Mdr2^−/−^/p19^ARF−/−^ and Stat3^Δhc^/Mdr2^−/−^mice is accompanied by hepatocellular proliferation and apoptosis. Representative 60x histological sections of livers from control or 2 or 7 weeks-old Mdr2^−/−^/p19^ARF−/−^ *(A)* and Stat3^Δhc^/Mdr2^−/−^mice *(B)*: Ki67 (upper row) and cleaved caspase-3 (lower row). Inset: quantification of positive cells for each marker. Data represent the mean ± SEM of the number of positive cells in at least ten different fields. Student's t test calculated versus control sections (*p<0.05; **p<0.01;***p<0.001).

Mdr2^−/−^ mice represent a widely used model for experimental liver fibrosis [16], [17] and are characterized by chronic liver injury and large periductal accumulation of MFBs. Similarly, Mdr2^−/−^/p19^ARF−/−^ double null mice [15] displayed a fibrotic phenotype comparable to Mdr2^−/−^ mice which allows, on one hand, investigation of *in vivo* fibrosis development and, on the other hand, isolation of MFBs for *in vitro* experiments that become immortalized upon loss of p19^ARF^, a gene involved in the negative control of cell cycle [15]. As observed in Mdr2^−/−^ mice, Mdr2^−/−^/p19^ARF−/−^ mice developed spontaneous fibrosis characterized by periductal accumulation of collagen and MFBs, as well as an increased number of Kupffer cells (F4/80 positive) (Suppl. Fig. 1). Importantly, these periductal changes were accompanied by damage of the hepatic parenchyma and compensatory hepatocyte proliferation, since we could not only detect increased apoptosis by cleaved caspase-3, but also increased numbers of Ki67 positive cells, (Fig. 1A). Fibrotic cells, easily recognized by their elongated form, condensed nuclei and positive expression for alpha-Smooth Muscle Actin (α-SMA) (Suppl. Fig. 1), stained for Ki67, but not for apoptosis. Of note, dying hepatocytes were mostly detected in the surrounding tissue of these fibrotic areas. Stat3^Δhc^/Mdr2^−/−^ mice represent a mouse model with conditional inactivation of Stat3 in hepatocytes and cholangiocytes of Mdr2^−/−^ mice [18]. Loss of the hepatoprotective transcription factor Stat3 strongly aggravated liver injury and fibrosis of the Mdr2^−/−^ fibrotic phenotype (Suppl. Fig. 2) leading to premature lethality. The compensatory hepatocyte proliferation due to parenchymal liver damage was severely reduced in this model (Suppl. Fig. 2, Fig. 1B).

**Figure 2:**
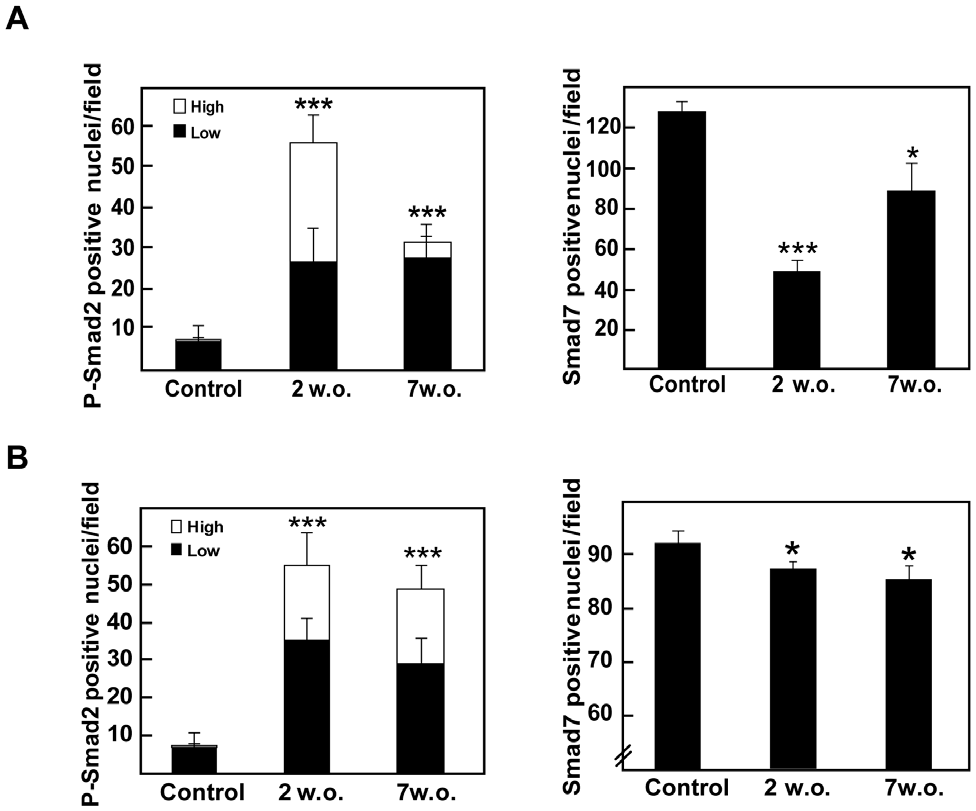
TGF-β pathway is activated at early stages of fibrosis in Mdr2^−/−^/p19^ARF−/−^ and Stat3^Δhc^/Mdr2^−/−^ mice. 60x histological sections of livers from control or 2 or 7 weeks-old Mdr2^−/−^/p19^ARF−/−^ *(A)* and Stat3^Δhc^/Mdr2^−/−^ mice *(B)* were stained with phospho-Smad2 or Smad7 and the number of positive cells was quantified. Left graph: positive nuclei for phospho-Smad2 with low or high signal intensity. Right graph: positive nuclei for Smad7. Data represent the mean ± SEM of cells in at least ten different fields. Student's t test calculated versus control sections (*p<0.05; **p<0.01;***p<0.001).

**Figure 3:**
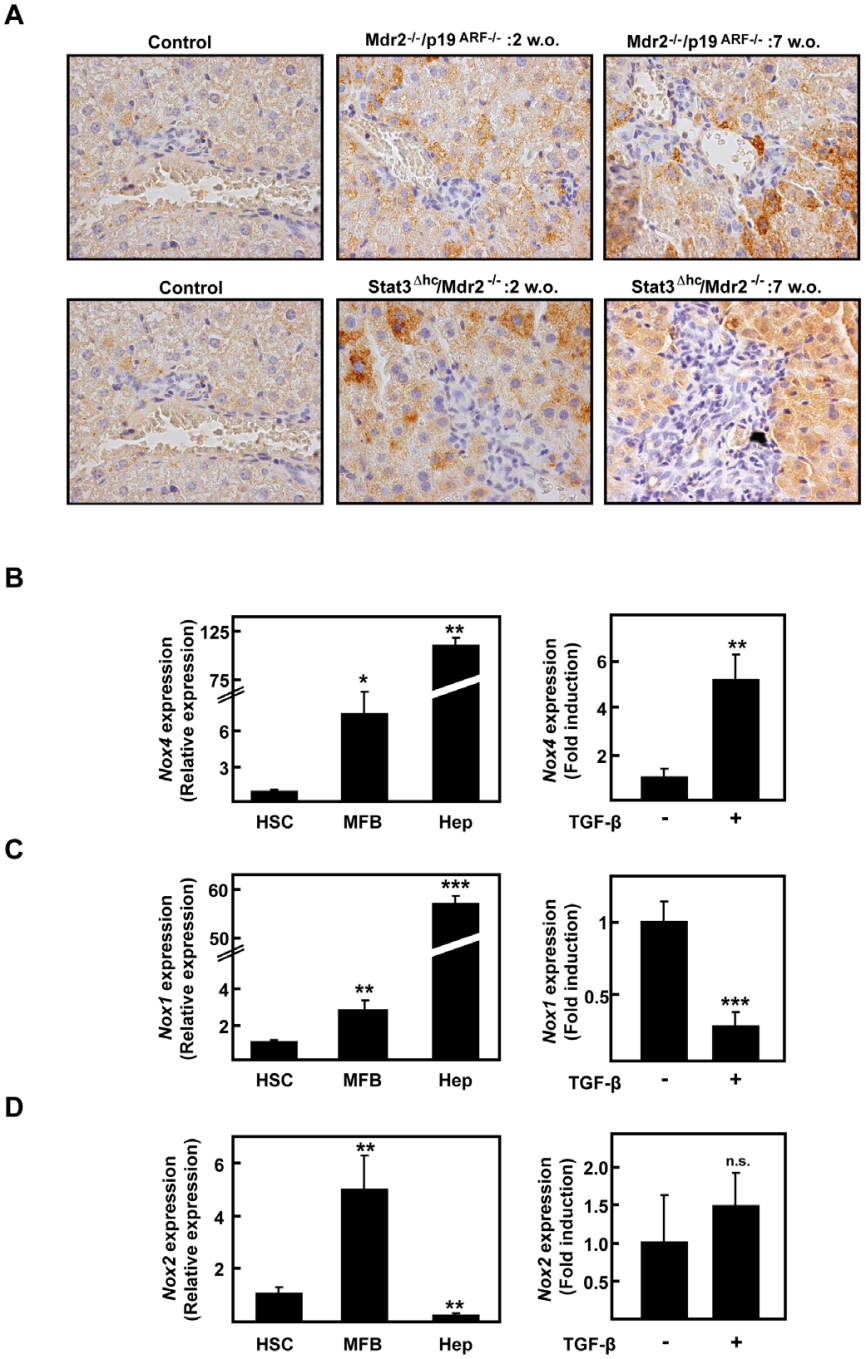
NOX4 expression is increased concomitant with fibrosis development in Mdr2^−/−^/p19^ARF−/−^ and Stat3^Δhc^/Mdr2^−/−^ mice. (*A*) Representative 60x photographs from NOX4 immunohistochemistry performed in control or 2 or 7 weeks-old livers. *(B*, *C*, *D)* Real time PCR of *NOX4, NOX1* or *NOX2. Left*: comparison of cultures of inactive stellate cells (HSC) isolated from p19^ARF−/−^ mice, *in vivo* activated myofibroblats (MFB) isolated from fibrotic Mdr2^−/−^/p19^ARF−/−^ livers and wild type immortalized hepatocytes (Hep) (see Materials and Methods section). *Right:* immortalized hepatocytes treated or not with 2 ng/ml TGF-β. Data represent the mean ± SEM of three independent experiments, and were calculated relative to HSC (left) or untreated hepatocytes (right) respectively, which were given an arbitrary value of 1. Student's t test calculated versus HSC cells in the left column (*p<0.05; **p<0.01) or untreated hepatocytes in the right column (**p<0.01).

Previous reports have indicated that several profibrotic genes are up-regulated at early stages of fibrosis development in Mdr2^−/−^ mice, highlighting the major pro-fibrotic cytokine, TGF-β [24]. In agreement with these previous results, we found that both TGF-β and its downstream signal molecule, phospho-Smad2, were increased during fibrosis development in both animal models as analyzed by immunohistochemistry (Fig. 2 and Suppl. Fig. 3). Phospho-Smad2 staining intensity was higher at 2 weeks and decreased over time, inversely correlating with Smad7 level, a TGF-β pathway inhibitor. Importantly, NOX4 level was also found elevated in these fibrosis models in both hepatocytes and fibroblastoid cells. In the case of hepatocytes, NOX4 expression was more intense in those cells surrounding the MFBs area (Fig. 3A), which was coincident with the regions showing positive cells for cleaved caspase-3 (Suppl. Fig. 4). Interestingly, these observations were corroborated in a model of chemically-induced fibrosis by CCl_4_ injection (Suppl. Fig. 5). CCl4 model has been widely used as an experimental model of chronic damage to the liver that produces fibrogenesis and may mimic the situation of human chronic liver diseases. These data together suggest that changes in the expression of NOX4 occur in different experimental animal models of hepatic fibrosis.

Since lack of p19^ARF^ allows the culture of spontaneously immortalized cells, we isolated and cultured both HSC in an inactive state from p19^ARF−/−^ non fibrotic livers and activated MFB from Mdr2^−/−^/p19^ARF−/−^ fibrotic livers, as described in the Materials and Methods section. These MFB, which have suffered the activation process *in vivo* during spontaneous fibrosis development in Mdr2^−/−^/p19^ARF−/−^mice, showed increased expression of NOX1, NOX2 and NOX4 at the mRNA level when compared to p19^ARF−/−^ inactive HSC (Fig. 3B–D, left graphs). Thus, these results suggest that these NOX isoforms may be induced during the HSC activation process. In addition, and corroborating the results at the tissue level, immortalized hepatocytes showed very high NOX4 expression when compared to HSC or MFB, which was further up-regulated when they were treated with TGF-β (Fig. 3B, right graph). NOX1 expression was also predominantly expressed in hepatocytes, but was down-regulated by TGF-β in *in vitro* experiments (Fig. 3C, right graph). NOX2 was predominantly expressed in MFBs and was not affected by TGF-β in hepatocytes (Fig. 3D).

### Role of NOX4 in HSC activation and MFBs phenotype maintenance

**Figure 4:**
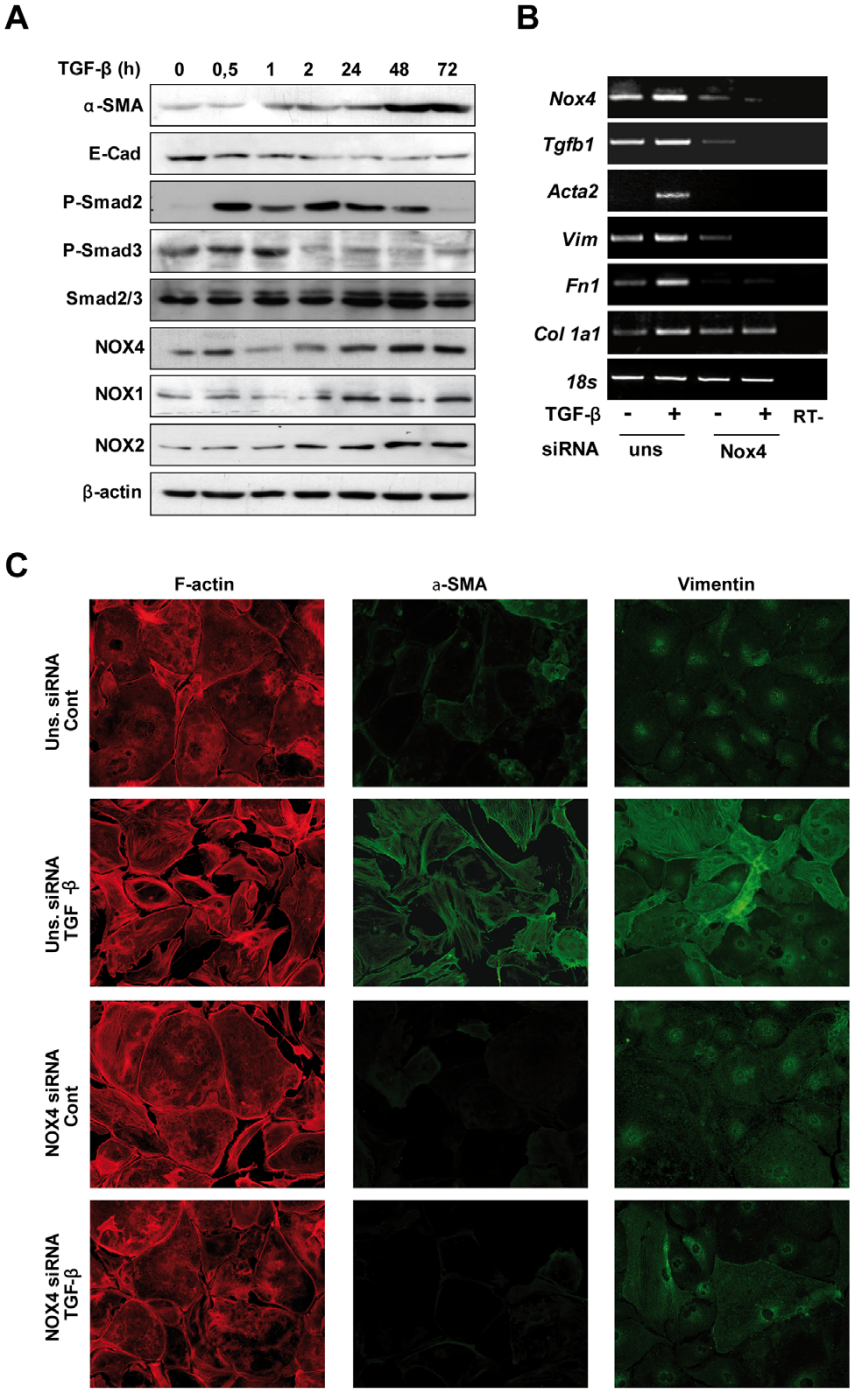
NOX4 knock-down inhibits TGF-β-dependent HSC activation. (*A*) p19^ARF−/−^ HSC cells at early passages were treated for the indicated times with 2 ng/ml TGF-β and a Western blot of total extracts was performed. β-actin was used as loading control. Additional information to this experiment is presented in Suppl. Fig. 6, where the expression of the different proteins in HSC cultured for 24, 48 and 72 h in the absence of treatments is shown, as well as densitometric analysis of the Western blots. In (*B*) and (*C*), p19^ARF−/−^ HSC cells were transfected with either an unsilencing siRNA (uns siRNA) or a specific siRNA for NOX4 (NOX4 siRNA) and then treated or not with 2 ng/ml TGF-β during 48 hours, when it was performed: (*B*) RT-PCR of *NOX4*, α-SMA (*Acta2*), vimentin (*vim*), fibronectin (*Fn1*) and collagen I (*Col1a1*); (*C)* immunofluorescence images of F-actin, α-SMA and vimentin. A representative image for each RT-PCR *(B)* or immunofluorescence staining *(C)* is shown. *18S* was used as loading control in *B*. Since HSC are very sensitive to serum deprivation, all experiments were carried out in the presence of 10% FBS.

Since NOX4 seemed to increase during the transdifferentiation process of HSC to MFBs and its role in this process is completely unknown, we decided to focus our study on the potential role of NOX4 in the *in vitro* activation of HSC with TGF-β. As expected, TGF-β treatment induced HSC activation, featured by increase in α-SMA levels and E-cadherin down-regulation, which was accompanied by NOX1, NOX2 and NOX4 up-regulation (Fig. 4A). In the absence of TGF-β, this activation was not observed (Suppl. Fig. 6). Regulation of α-SMA at the protein level correlated with parallel changes at the mRNA level, along with up-regulation of vimentin and the extracellular matrix genes collagen I and fibronectin (Fig. 4B). The same panel shows that all these changes in gene expression induced by TGF-β were inhibited when NOX4 was knocked-down in cultured HSC cells. Importantly, phenotypical changes induced by TGF-β during the transdifferentiation process, such as morphological alterations and increased expression and reorganization of α-SMA and vimentin, were impaired when NOX4 was targeted knock-down (Fig. 4C). No apparent changes in the viability of these cells were observed in NOX4 deficient cells (results not shown).

**Figure 5:**
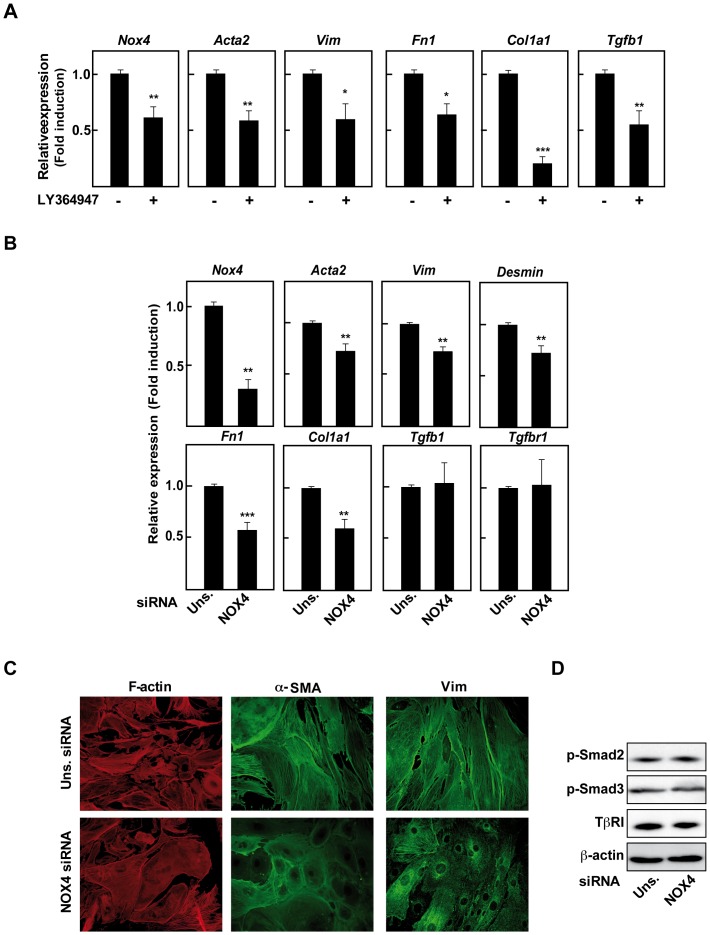
NOX4 downstream autocrine TGF-β is necessary to maintain the activated phenotype of cultured Mdr2^−/−^/p19^ARF−/−^ **MFB.** MFB isolated from fibrotic Mdr2^−/−^/p19^ARF−/−^ livers were used. *(A)* Real time PCR of Mdr2^−/−^/p19^ARF−/−^ MFB treated for 72 hours with 10 μM LY36494 (TRβI inhibitor): *NOX4*, α-SMA (*Acta2*), vimentin (*vim*), fibronectin (*Fn1*), collagen I (*Col1a1*) and TGF-β_1_ (*Tgfb1*). In *B–D*, Mdr2^−/−^/p19^ARF−/−^ MFB were transfected with either an unsilencing siRNA (uns siRNA) or a specific siRNA for NOX4 (NOX4 siRNA) for 72 hours, when it was performed: *(B)* Real time PCR of the indicated genes. *(C)* Immunofluorescence staining of F-actin, α-SMA and vimentin. *(D)* Western blot of total lysates. β-actin was used as loading control. Data represent the mean ± SEM of three to six independent experiments, and were calculated relative to untreated MFB *(A)* or unsilencing-transfected cells *(B)* (*p<0.05; **p<0.01).

Since TGF-β and NOX4 appeared to play a crucial role in the activation of HSC, we next wondered if they could play a role in the maintenance of the activated MFB phenotype. For this purpose, firstly we treated isolated Mdr2^−/−^/p19^ARF−/−^ MFBs during 72 hours with LY364947, an inhibitor of the TGF-β receptor I (TβRI), in *in vitro* experiments. As shown in Figure 5A, inhibition of the TβRI reversed the MFB phenotype, measured as expression of pro-fibrotic genes, which correlated with a decrease in NOX4 mRNA levels. Importantly, this fact implicates plasticity of the MFB phenotype suggesting that it is possible to reverse the MFB activated state towards a more inactive HSC-like phenotype. It is worthy to note that blocking the TGF-β pathway down-regulated TGF-β expression, indicating the existence of an autocrine positive feed-back loop implicated in the maintenance of the MFB properties. In addition, as shown in Fig. 5B, NOX4 knock-down produced a similar change as the one found with the TβRI inhibitor. Indeed, the expression of pro-fibrotic genes, as well as α-SMA and vimentin expression and organization, were significantly diminished when NOX4 was knocked-down, cells acquiring an HSC-like morphology (Fig. 5C). Furthermore, desmin expression, as a marker of activated stellate cells, decreased when NOX4 was knocked-down, correlating with the reversion of MFB phenotype. However, NOX4 silencing was unable to inhibit the expression of either TGF-β or its receptor (Fig. 5B, 5D), and it did not alter Smad2/3 phosphorylation status (Fig. 5D).

These results together suggest that NOX4 acts downstream TGF-β and controls the expression of different pro-fibrotic genes; however, autocrine expression of the cytokine in MFB, and activation of its downstream immediate signals, i.e., Smads, seems to be independent of NOX4.

It is interesting to point out that NOX4 expression at the mRNA level in both HSC and MFB was much higher than the expression of the other isoforms NOX1 and NOX2 (50-fold higher than NOX2 and 25-fold higher than NOX1 in hepatocytes; 80-fold higher than NOX2 and 60-fold higher than NOX1 in HSC: Suppl. Fig. 7), which may explain why neither NOX1 nor NOX2 replaces NOX4 function.

### Role of NOX4 in hepatocytes

**Figure 6:**
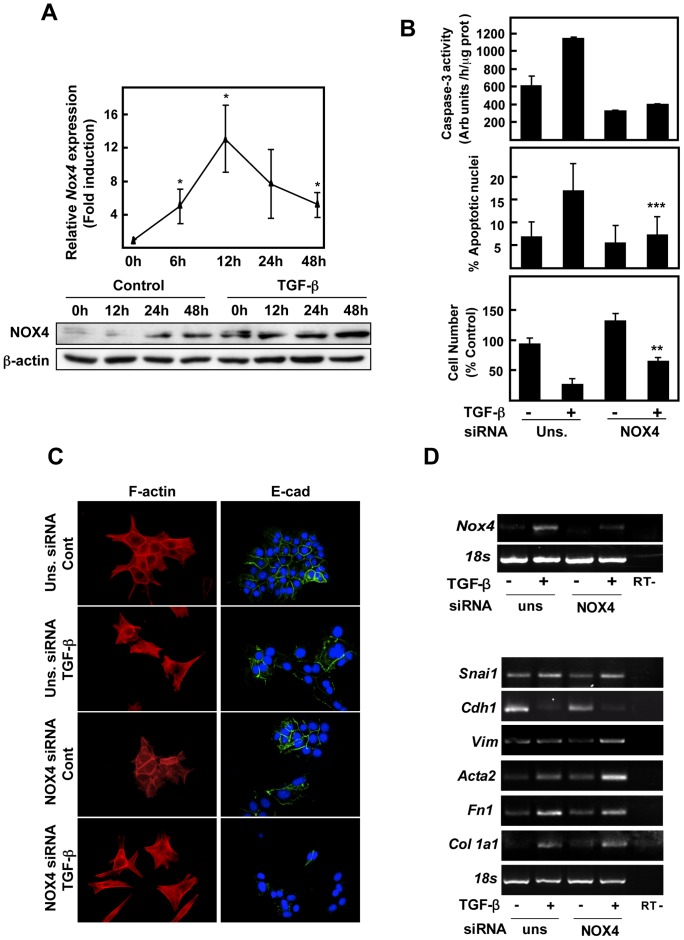
NOX4 is required for apoptosisbut not necessary for the EMT process, induced by TGF-β in hepatocytes. *(A)* TGF-β induces NOX4 expression in hepatocytes. NOX4 transript and protein levels were determined by Real time-PCR (upper panel) and western blot (lower panel), respectively, at the indicated times of treatment with 2 ng/ml TGF-β. In *B–D*, immortalized hepatocytes were transfected with either an unsilencing siRNA (uns siRNA) or a specific siRNA for NOX4 (NOX4 siRNA), serum-depleted for 4 hours and, finally, treated or not with 2 ng/ml TGF-β. *(B)* Cell death analysis: activation of caspase-3 activity at 16 hours, where the peak of maximal activation was found *(upper graph)*; percentage of cells with apoptotic nuclei at 48 h upon nuclear staining with DAPI *(middle graph)*; loss in cell viability at 48 h *(lower graph)*. Data represent the mean ± SEM of three independent experiments. Student's t test calculated comparing TGF-β-treated cells between the two conditions with the different siRNAs (**p<0.01; ***p<0.001). *(C)* Fluorescence microscopy staining for F-actin and E-cadherin at 48 hours. *(D)* Representative RT-PCR of the indicated genes after 3 h of treatment to observe the effects on *Nox4* expression (upper panel) and 48 h of treatment to analyze the effects on EMT-related genes (bottom panel). *18S* was used as loading control.

Finally, we decided to study the putative role of NOX4 in TGF-β-induced effects in hepatocytes. As shown in Fig. 6A, NOX4 expression was significantly up-regulated reaching the maximum mRNA levels at 12 h and the maximum protein level at 24–48 hours upon TGF-β treatment. As we and others have previously reported in several experimental models, induction of apoptosis by TGF-β was impaired when NOX4 was knocked-down (Fig. 6B). These *in vitro* data support the immunohistochemistry studies, where elevated NOX4 expression seemed to correlate with the areas of higher apoptosis of hepatocytes (Suppl. Fig 4). Interestingly, supplementation of cell culture medium with either antioxidants or a general inhibitor of NADPH oxidases (DPI) blocked TGF-β-induced ROS production and caspase-3 activation in hepatocytes (Suppl. Fig. 8A, B). In a similar way, a permeable form of GSH and DPI also attenuated changes in gene expression addressed by TGF-β in HSC (Suppl. Fig. 8C), highlighting the relevant role played by ROS in these processes.

**Figure 7:**
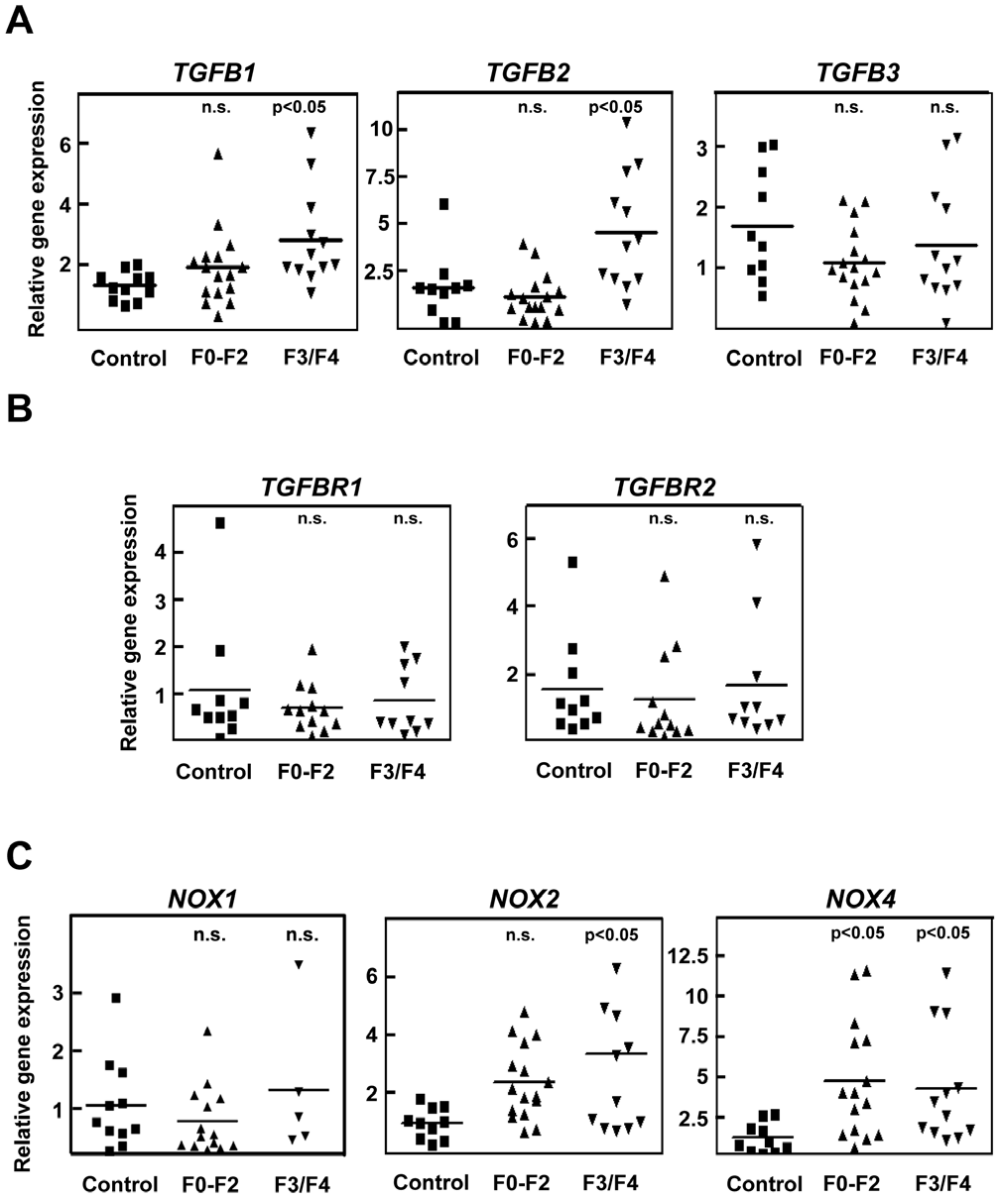
TGF-β1 and 2, NOX2 and NOX4 are up-regulated in human samples from patients with HCV-derived fibrosis. Samples from patients showing control livers (control; n = 10), low or mildly fibrotic livers (F0–F2; n = 16) or severely fibrotic and cirrhotic livers (F3/F4; n = 12). Real time determination of TGF-β_1_(*TGFB1*), TGF-β_2_ (*TGFB2*), TGF-β_3_ (*TGFB3*) (*A*), TGFβRI and TGFβRII (*TGFBR1*, *TGFBR2*) (*B*), and *NOX1*, *NOX2* and *NOX4* (*C*). Statistic was calculated using one-way ANOVA associated with Dunnett's test.

**Table 1:** Demographic, virological and histopathological characteristics of patients with chronic hepatitis C.

Age (yr) (Median and range)		46 (31–67)
Gender (M/F)		17/11
Baseline Viral load (×10^3^ IU/ml) (Mean ± SD)		1.36±2.05
Genotype distribution (%)	Ia	7.14
	Ib	75.00
	II	3.57
	III	14.28
Activity distribution (n, %) (METAVIR)	0	0 (0)
	1	11 (39.28)
	2	14 (50.00)
	3	3 (10.71)
	4	0 (0)
Fibrosis distribution (n, %) (METAVIR)	0	1 (3.57)
	1	11 (39.28)
	2	4 (14.28)
	3	7 (25.00)
	4	5 (17.85)

Tissue of liver biopsies from 28 patients with chronic hepatitis C in different stages of liver fibrosis was examined for the gene expression analysis shown in Fig. 7.

We have previously reported that some liver cells are able to impair the pro-apoptotic effects of TGF-β and undergo EMT, characterized by cytoskeleton rearrangement and changes in gene expression leading to a mesenchymal phenotype with up-regulation of Snail, vimentin and α-SMA and loss of E-cadherin expression [21], [25]. In view of the results regarding apoptosis, we next wondered whether NOX4 could be mediating TGF-β-induced EMT *in vitro*. NOX4 knock-down did not influence TGF-β-induced cytoskeletal changes, neither affected the expression of several EMT-related genes (Fig. 6C, D). In summary, NOX4 is implicated in apoptosis but not in the EMT process that TGF-β induces in hepatocytes.

### Up-regulation of TGF-β and NOX4 in human samples from HCV infected patients

Since our *in vivo* and *in vitro* results in animal models pointed to a crucial role for NOX4 in fibrosis development, we next studied the situation in human samples. For this reason, we chose patients suffering from different degrees of liver fibrosis associated to HCV infection, who were classified as having mildly fibrotic livers (F0–F2) or severely fibrotic and cirrhotic livers (F3/F4) (Table 1). Comparing with samples from control livers, we performed real time PCR determinations of the three isoforms of TGF-β and the corresponding receptors, and also NOX1, NOX2 and NOX4. As shown in Fig. 7, TGF-β1 shows a clear tendency to increase from F0/F2 stages, showing significant enhanced levels in F3/F4 patients. TGF-β2 was also significantly increased in the F3/F4 stage (Fig. 7A). No relevant changes in the expression of their receptors were observed. Interestingly, NOX1 expression did not show any significant change (Fig. 7C, left graph), but NOX2 and NOX4 were significantly up-regulated in fibrotic livers, being the relative increase in NOX4 higher than that observed for NOX2 (Fig. 7C).

## Discussion

Chronic liver disease often progresses to fibrosis and finally to cirrhosis, which is a preneoplastic condition [1]. Thus far, there are no direct therapies aimed at liver fibrosis reversal, therefore innovative antifibrogenic approaches are needed [26]. Different models of hepatic fibrosis have been used to study the molecular pathogenesis of this disease. From these studies, several key generalizations have been done [1]: i) TGF-β is the most potent liver pro-fibrogenic cytokine; ii) oxidative stress induces liver fibrosis; iii) blocking normal liver regeneration by massive hepatocyte apoptosis turns out to be pro-fibrogenic. One of the most studied mechanisms of fibrogenesis actually influenced by ROS is myofibroblast activation. Previous reports and results presented in this manuscript have revealed that stellate cell transdifferentiation into myofibroblast is inhibited by antioxidants [27], [28]. NOX4 downstream TGF-β has been described as the main mediator for myofibroblast activation in different organs such as heart [12], lung [11], kidney [13] and diseased prostatic stroma [14]. However, very few were known about the role of NOX4 in liver fibrosis. Results presented here indicate that induction of NOX4 occurs in three different animal models of liver fibrosis and in chronic HCV infection in humans, associated with activation of the TGF-β pathway, appearance of fibrotic areas and hepatocyte proliferation and apoptosis. NOX4 may play a key role in liver fibrosis development, downstream TGF-β, at two different levels: i) *in vitro* experiments reveal that NOX4 is required for both HSC activation and maintenance of the activated phenotype in MFBs and ii) hepatocytes respond to TGF-β by inducing NOX4 that is required for its pro-apoptotic response, which might be relevant to blunt regeneration and create a pro-fibrogenic microenvironment.

However, the role of NOX proteins in liver fibrogenesis is not only circumscribed to NOX4. Thus, studies performed in Nox1^−/−^, Nox2^−/−^ or p47phox^−/−^ mice have pointed out the importance of NOX1 and NOX2 in fibrosis development [5], [29], [30]. Our results indicate that expression of NOX4 at the mRNA levels is much higher than those found for NOX1 and NOX2 in HSCs and hepatocytes, and functions are not redundant, since knock-down of NOX4 in these cells cause effects that cannot be prevented by the other NOXes. It is possible that each isoform plays differential roles in liver fibrosis. Indeed, it has been suggested that NOX1 promotes myofibroblast proliferation by PTEN inactivation to positively regulate an Akt/FOXO4/p27 signaling pathway [29]. NOX2, highly expressed in macrophages and bone marrow-derived cells [8] may be acting in the process of phagocytosis of dead hepatocytes [30]. NOX1 activity might also contribute to the inflammatory process promoting COX-2 expression and prostaglandin synthesis in hepatocytes [31]. Interestingly, whereas the revised version of this manuscript was being prepared, evidences for the role of dual NOX4/NOX1 pharmacological inhibitors in decreasing both the apparition of fibrogenic markers and hepatocyte apoptosis *in vivo*, upon bile duct ligation, were reported [32], highlighting the relevant role of NOX1 and NOX4 in liver fibrosis and opening new perspectives for its treatment.

Hepatic fibrosis has been considered as an irreversible process but recent experimental and clinical data indicate that removal of the pro-fibrotic agent or condition may reverse liver fibrosis [33]. Results presented in this manuscript suggest that either TβRI inhibition or NOX4 silencing reverses the MFB phenotype, decreasing the expression of extracellular matrix genes, such as collagen I or fibronectin, down-regulating α-SMA and vimentin expression and changing cell morphology, which loses myofibroblastic appearance. Since it has been recently proposed that NOX4 modulates α-SMA and procollagen I (alpha1) expression in pulmonary fibrosis by controlling activation of Smad2/3 [34], we checked the effect of silencing NOX4 on TGF-β levels and Smad2/3 phosphorylation. Our results have indicated that NOX4 clearly acts downstream TGF-β-independently from Smads activation. It also has been described that NOX4 is critical for maintenance of smooth muscle gene expression in vascular smooth muscle cells [35], where ROS production impairs the TGF-β-induced phosphorylation of Ser103 on serum response factor (SRF) and reduces its transcriptional activity. Thus, NOX4 may play an important role associated with the α-SMA phenotype, being not only important in fibrotic processes, but also in cardiovascular physiology.

We have previously described that liver cells respond to TGF-β *in vitro* undergoing EMT [25], [36]. The role of EMT is perhaps the most intriguing and controversial of recent hypothesis on the origin mechanisms of liver fibrosis [37]. Strong evidences indicate that hepatocytes from transgenic animals that overexpress Snail (a master gene involved in EMT through its capacity to repress E-cadherin gene, among others) fully undergo EMT [38] and may propagate liver fibrosis progression [39]. However, under normal genetic background, data from different experimental approaches in animals and humans show controversy [40]. Our results show that EMT occurs in hepatocytes *in vitro*, but NOX4 is not required for this process. However, NOX4 mediates TGF-β-induced cell death that is prevented in the presence of antioxidants. In agreement with these results, it has been recently proposed a role for NOX4 in epithelial cell death during development of bleomycin-induced lung fibrosis [41]. Using a model of NOX4-deficient mice, authors demonstrated that these animals were resistant to fibrosis due to the abrogation of TGF-β-induced apoptosis in epithelial cells. Prevention of apoptosis impaired fibrosis development, although inflammation was comparable to wild-type. A similar situation may occur in liver fibrosis, where engulfment of apoptotic bodies by HSC contributes to induce their activation [7]. Indeed, hepatocyte apoptosis not only would facilitate fibrosis through blocking liver regeneration, but it could play an active role. In this line of evidence, inhibiting apoptosis decreased the liver profibrogenic response [7], [42]. Additionally to the crucial role of NOX4 in TGF-β-induced cell death, recent results indicate that it may be also required for apoptosis induced by other stimuli in liver cells, such as FasL and TNF-α/actinomycin D [32].

Finally, the finding that NOX4 is induced during the progression of a HCV disease reinforces the hypothesis of a role for NOX4 in human liver fibrosis. The magnitude of NOX4 up-regulation is higher than that observed for its co-partner NOX2 and, interestingly, we could not find any significant change in the expression of NOX1. NOX4 induction is observed at early stages of the disease when increases of TGF-β1 and 2 are not significant yet. This could be mediated by release of inflammatory signals that, indeed, up-regulate NOX4 in hepatocytes [31]. Furthermore, different reports support that HCV induces a persistent elevation and increased nuclear localization of NOX4 in *in vitro* assays in hepatocytes, a process that was TGF-β-dependent [43], [44]. Collectively, all these data provide evidences to propose that HCV-induced NOX4 may contribute to ROS production and may be related to HCV-induced liver disease. Results presented in this manuscript support that NOX4 could play an essential role inducing activation of stellate cells and apoptosis of hepatocytes under these conditions of human disease, contributing to the development of liver fibrosis.

Development of first-in-class series of NOX4 inhibitors for the potential treatment of fibrotic diseases, cardiovascular and metabolic syndromes is in progress [45]. Liver fibrosis might be considered for future clinical trials with these drugs. Likewise, ROS and NOX4 induced by TGF-β have proved to be therapeutic targets of polyenylphosphatidylcholine in the suppression of human stellate cell activation [46]. Since NOX4 is mainly expressed in hepatocytes and HSC [8], according to the results presented in this manuscript, NOX4 inhibitors would specifically prevent HSC activation and hepatocytes cell death, without altering the role of other NOXes, such as NOX2, which might play defense function in Kupffer cells. In advanced stages of the disease, NOX4 inhibitors might be able to reverse the fibrotic phenotype acting on MFBs. Furthermore, and not less important, we demonstrate that silencing NOX4 prevents fibrogenesis but has no effect on TGF-β-mediated Smads phosphorylation. Indeed, the use of pharmacological drugs targeting NOX4 expression/activation would inhibit fibrogenesis without blocking other beneficial effects of TGF-β, such as growth inhibition in the epithelial cells, which prevents initiation of a pre-neoplastic stage.

In summary, here we show that NOX4 expression is elevated in the livers of experimental in vivo models of liver fibrosis and in patients with chronic HCV-derived infection, increasing along the fibrosis degree. NOX4, downstream TGF-β pathway, would play a role in the acquisition and maintenance of the MFB phenotype, as well as in mediating death of hepatocytes, which provokes inflammation and facilitates extracellular matrix deposit.

## Supporting Information

Figure S1 **Fibrosis development in Mdr2^−/−^/p19^ARF−/−^ mice.** Representative histological sections of livers from control or 2 or 7 weeks-old Mdr2^−/−^/p19^ARF−/−^ mice. H&E, trichrome C staining and immunohistochemistru of F4/80, α-SMA, E-cadherin (E-cad) or vimentin (Vim) are shown.
(TIF)

Figure S2 **Fibrosis development in Stat3^Δhc^/Mdr2^−/−^ mice.** Representative histological sections of livers from control or 2 or 7 weeks-old Stat3^Δhc^/Mdr2^−/−^ mice. H&E, trichrome C staining and immunohistochemistru of F4/80, α-SMA, E-cadherin (E-cad) or vimentin (Vim) are shown.
(TIF)

Figure S3 **TGF-β pathway is activated in Mdr2^−/−^/p19^ARF−/−^ and Stat3^Δhc^/Mdr2^−/−^ mice.** Immunohistochemistry analysis of TGF-β, phospho-Smad2 or Smad7 in Mdr2^−/−^/p19^ARF−/−^ (**A**) and Stat3^Δhc^/Mdr2^−/−^ mice (**B**).
(TIF)

Figure S4 **NOX4 expression in hepatocytes correlates with activation of caspase-3 in fibrotic tissues from Mdr2^−/−^/p19^ARF−/−^ mice.** Immunohistochemistry analysis of NOX4 and the active form (cleaved) of caspase-3 in: hepatocytes around the vascular (up) or fibrotic (bottom) areas (**A**); two serial sections showing coincidence in the expression of both proteins in the same cells (**B**).
(TIF)

Figure S5 **NOX4 expression is increased concomitant with fibrosis development in CCl_4_ injection.** Liver samples were collected and processed for immunohistochemical analysis. Representative results for NOX4, cleaved caspase-3 and Ki67 are shown.
(TIF)

Figure S6 **Additional information to** **Figure 4** **.** Expression of the different proteins analyzed in the Western blot of Figure 4A in HSC cells cultures for 24, 48 and 72 h in the absence of treatments (**A**). Quantitation of the intensity of the bands through densitometric analysis, relative to loading (β-actin) is shown above each band. A similar quantification approach for Western blot in Figure 4A is shown in **B**.
(TIF)

Figure S7 **Role of NOX4 knock-down with specific siRNA on the expression of NOX4, NOX2 and NOX1 in HSC (A) and MFB (B).** p19^ARF−/−^ HSC cells (**A**) and Mdr2^−/−^/p19^ARF−/−^ MFB (**B**) were transfected with either an unsilencing siRNA (uns. siRNA) or a specific siRNA for NOX4 (NOX4 siRNA) as described in Figs. 4 and 5, respectively. In the case of HSC (**A**), cells were either not treated or treated with TGF-β (2 ng/ml), as indicated in the figure. Data shown correspond to the real-time PCR analysis of the mRNA levels of NOX4, NOX2 and NOX1, which were calculated relative to 18S expression. In order to compare the expression level among the different isoforms, we gave an arbitrary value of 1 to NOX4 expression under basal conditions in both HSC (**A**) and MFB (**B**), and all the data are referred to this value. Mean ± S.E.M. is shown and the specific value of the mean detailed above each bar.
(TIF)

Figure S8 **Role of antioxidants (GEE and BHA) or a NADPH oxidase inhibitor (DPI) on the effects of TGF-β on ROS production and apoptosis in hepatocytes (A and B, respectively) and activation of HSC (C).** Cells were pre-incubated during 30 min with 1 μM DPI, 2 mM GEE or 200 μM BHA, as indicated in each figure, before adding 2 ng/mlof TGF-β. The same concentrations of the agents were maintained during all the TGF-β treatment. **A.** After 3 h of treatment in hepatocytes, intracellular content of ROS was analyzed through fluorimetric assay as detailed in the Materials and Methods section. Data represent the mean ± S.E.M. of 3 independent experiments in triplicate and are referred to the value in untreated cells (100%). **B.** After 16 h of treatment in hepatocytes, proteins were collected for caspase-3 analysis. Data represent the mean ± S.E.M. of 3 independent experiments in duplicate and are expressed as arbitrary units per hour and per microgram of protein. **C.** After 48 h of treatment in HSC, RNA was collected for analysis of expression by RT-PCR of the genes detailed in the figure. A representative experiment out of 3 is shown.
(TIF)

Table S1 **Mouse primer sequences used for semiquantitative PCR.**
(DOC)

Table S2 **Mouse and human primer sequences used for quantitative real-time PCR.**
(DOC)
