# Supplementary figures and images for: NADPH Oxidase NOX4 Mediates Stellate Cell Activation and Hepatocyte Cell Death during Liver Fibrosis Development
Source: PLoS One. 2012 Sep 26;7(9):e45285. doi: 10.1371/journal.pone.0045285 (PMC3458844; doi:10.1371/journal.pone.0045285)

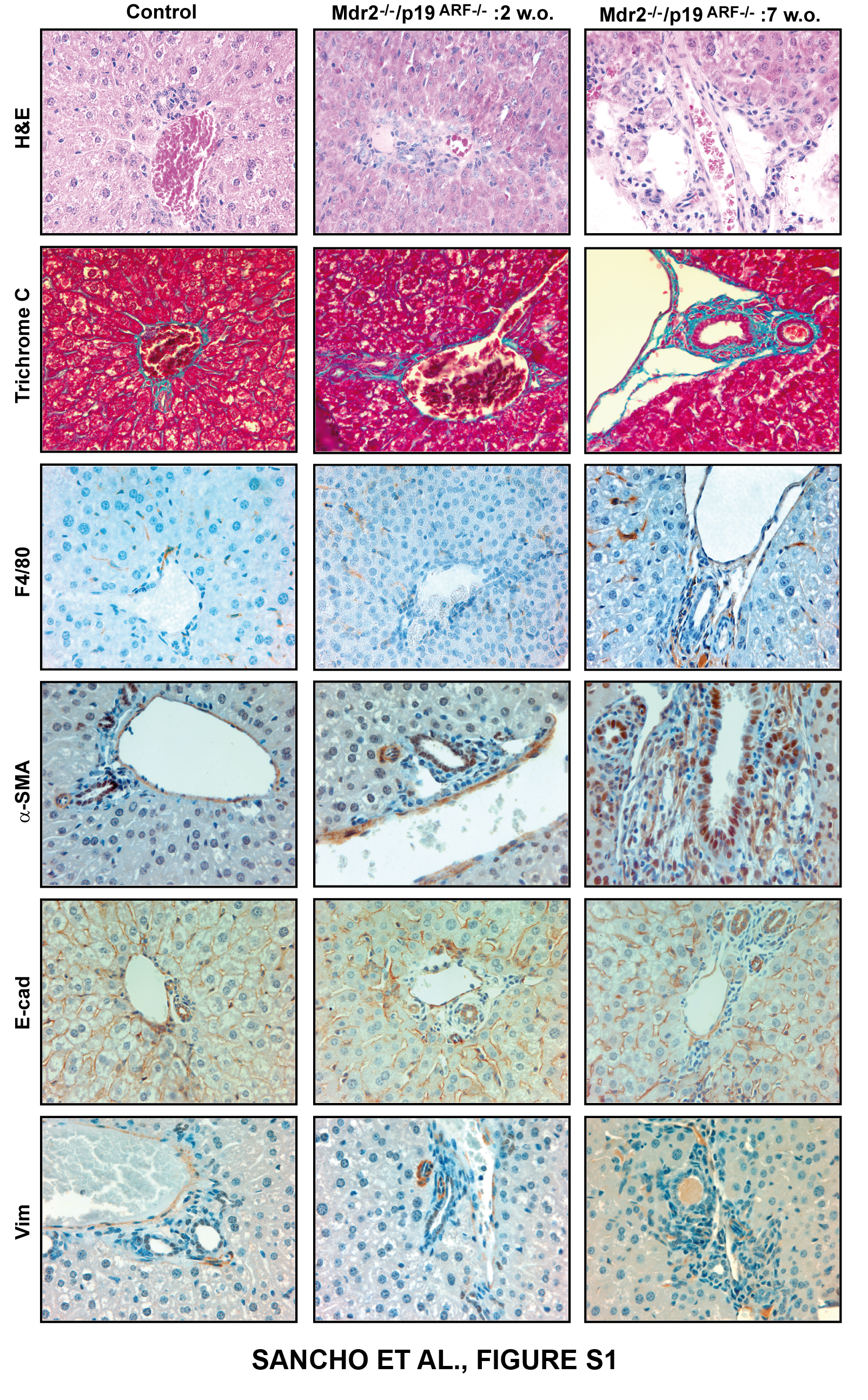

Supplement: Figure S1 — Fibrosis development in Mdr2−/−/p19ARF−/− mice. Representative histological sections of livers from control or 2 or 7 weeks-old Mdr2−/−/p19ARF−/− mice. H&E, trichrome C staining and immunohistochemistru of F4/80, α-SMA, E-cadherin (E-cad) or vimentin (Vim) are shown. (TIF) [file pone.0045285.s001.tif]

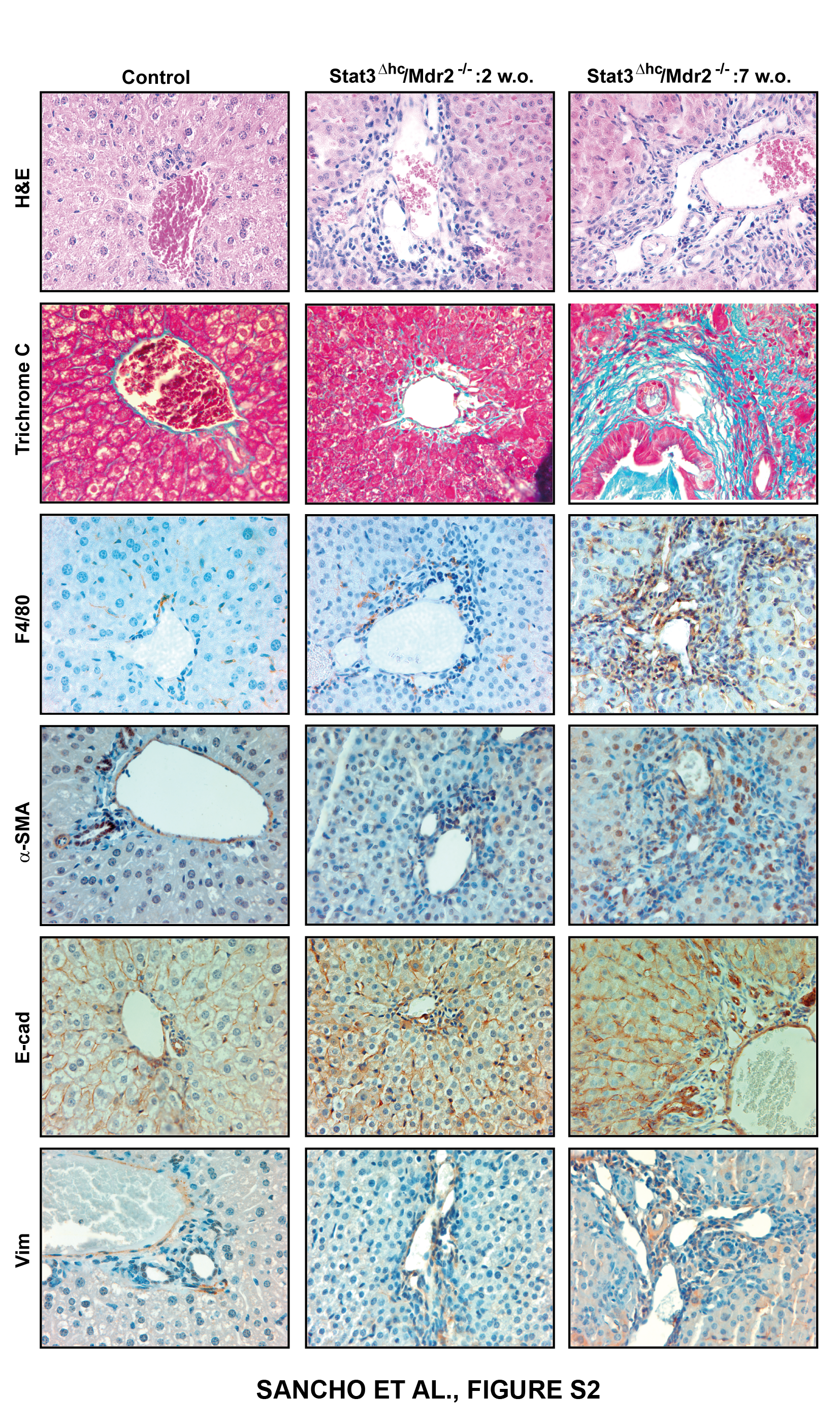

Supplement: Figure S2 — Fibrosis development in Stat3Δhc/Mdr2−/− mice. Representative histological sections of livers from control or 2 or 7 weeks-old Stat3Δhc/Mdr2−/− mice. H&E, trichrome C staining and immunohistochemistru of F4/80, α-SMA, E-cadherin (E-cad) or vimentin (Vim) are shown. (TIF) [file pone.0045285.s002.tif]

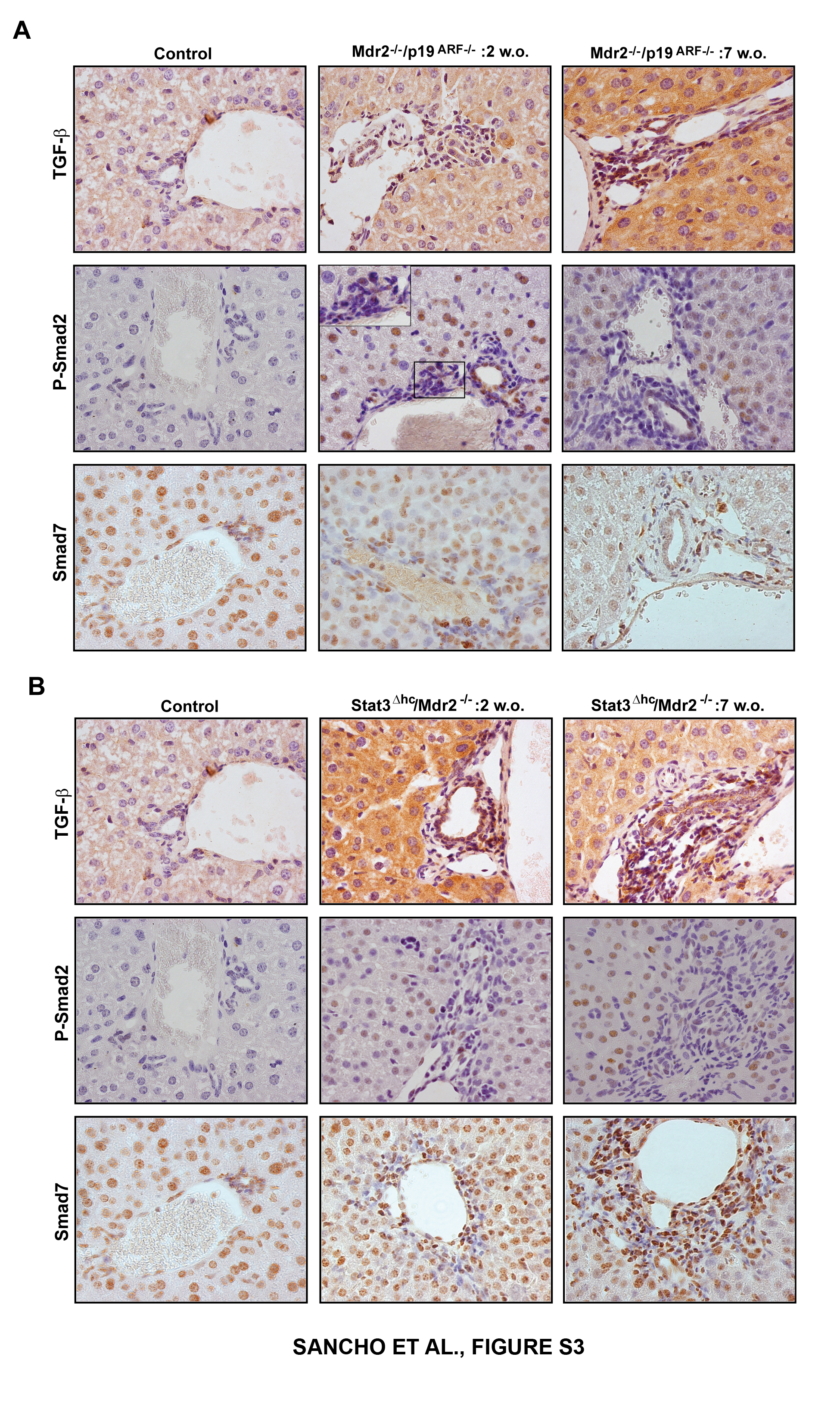

Supplement: Figure S3 — TGF-β pathway is activated in Mdr2−/−/p19ARF−/− and Stat3Δhc/Mdr2−/− mice. Immunohistochemistry analysis of TGF-β, phospho-Smad2 or Smad7 in Mdr2−/−/p19ARF−/− (A) and Stat3Δhc/Mdr2−/− mice (B). (TIF) [file pone.0045285.s003.tif]

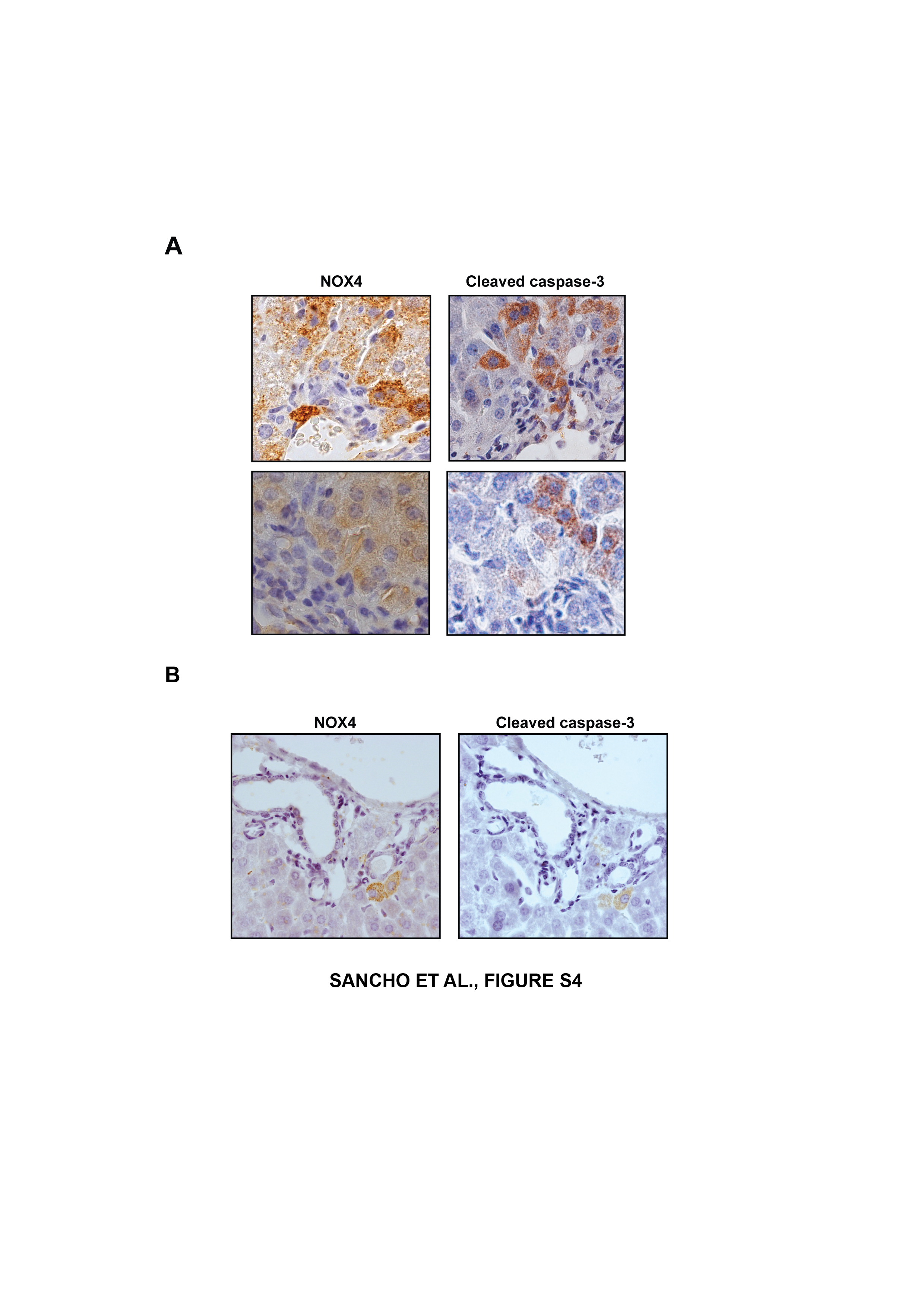

Supplement: Figure S4 — NOX4 expression in hepatocytes correlates with activation of caspase-3 in fibrotic tissues from Mdr2−/−/p19ARF−/− mice. Immunohistochemistry analysis of NOX4 and the active form (cleaved) of caspase-3 in: hepatocytes around the vascular (up) or fibrotic (bottom) areas (A); two serial sections showing coincidence in the expression of both proteins in the same cells (B). (TIF) [file pone.0045285.s004.tif]

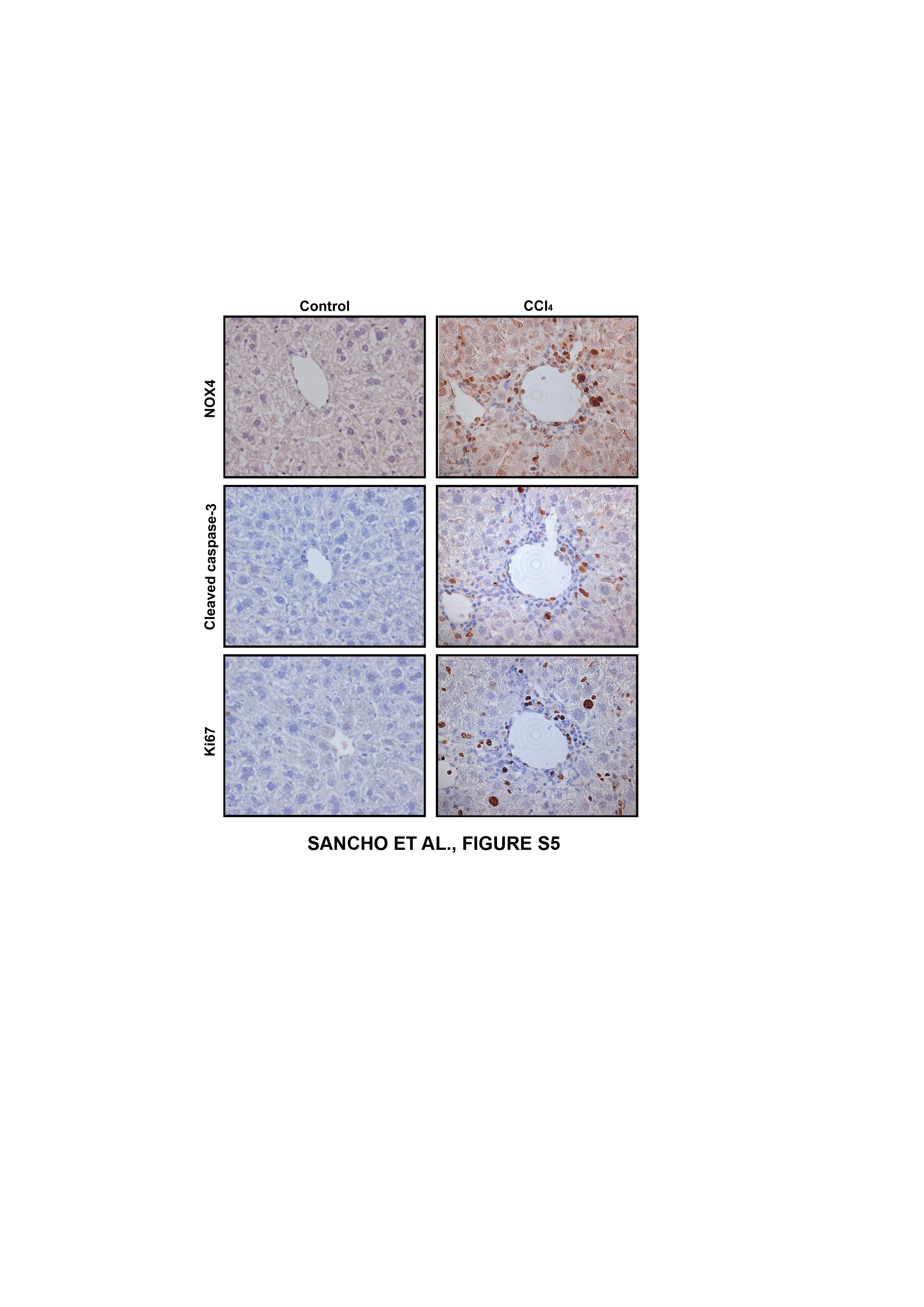

Supplement: Figure S5 — NOX4 expression is increased concomitant with fibrosis development in CCl4 injection. Liver samples were collected and processed for immunohistochemical analysis. Representative results for NOX4, cleaved caspase-3 and Ki67 are shown. (TIF) [file pone.0045285.s005.tif]

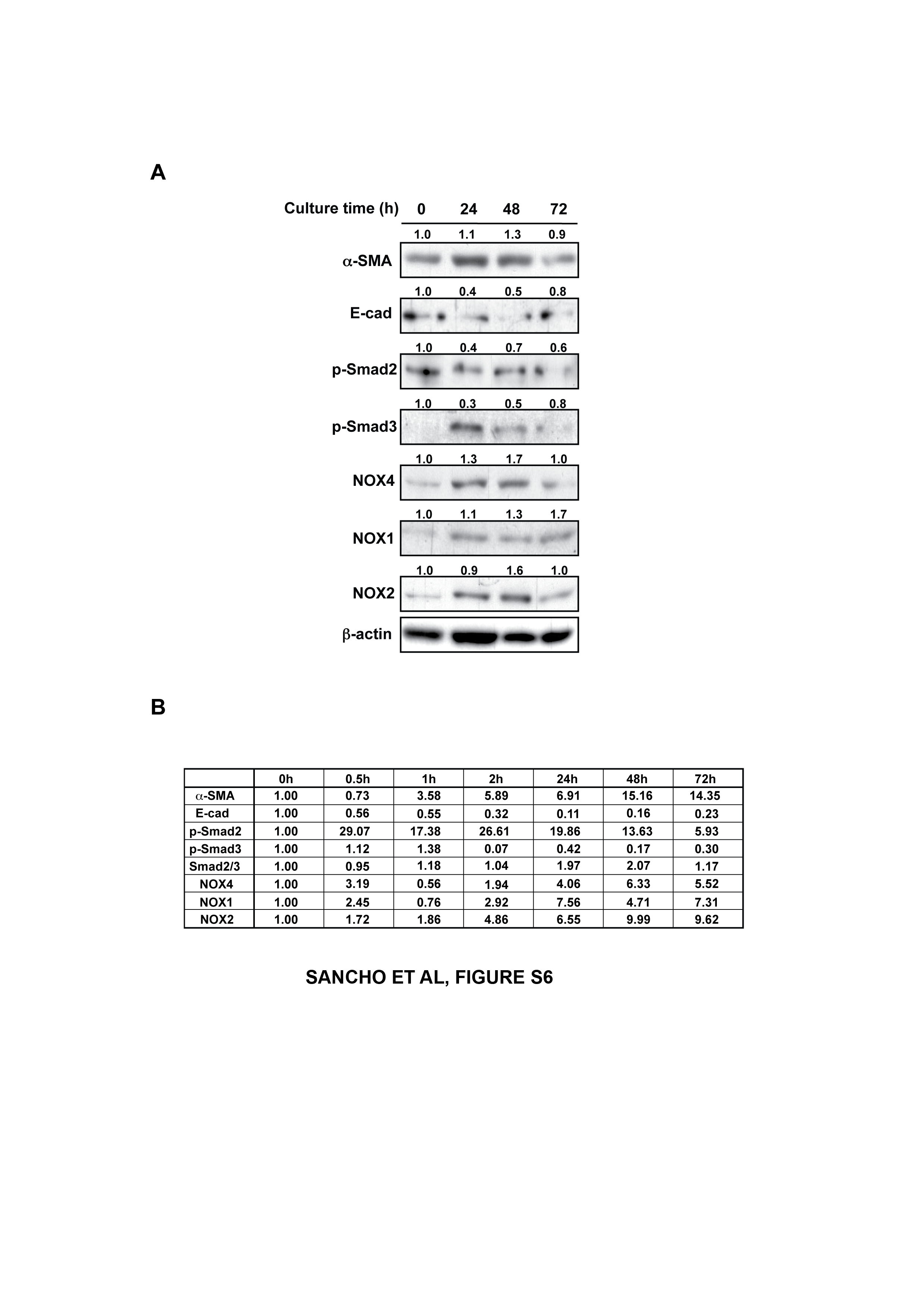

Supplement: Figure S6 — Additional information to Figure 4 . Expression of the different proteins analyzed in the Western blot of Figure 4A in HSC cells cultures for 24, 48 and 72 h in the absence of treatments (A). Quantitation of the intensity of the bands through densitometric analysis, relative to loading (β-actin) is shown above each band. A similar quantification approach for Western blot in Figure 4A is shown in B. (TIF) [file pone.0045285.s006.tif]

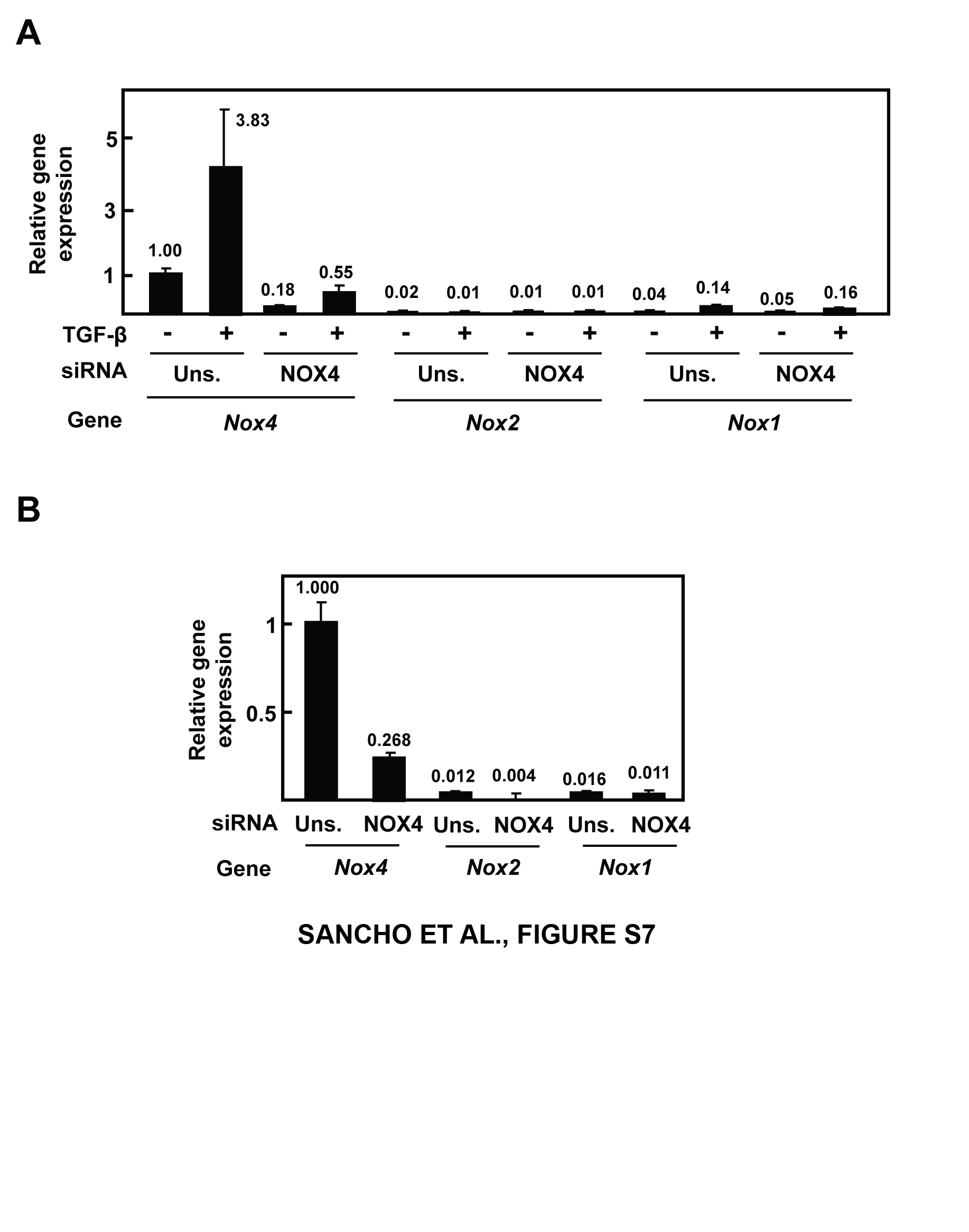

Supplement: Figure S7 — Role of NOX4 knock-down with specific siRNA on the expression of NOX4, NOX2 and NOX1 in HSC (A) and MFB (B). p19ARF−/− HSC cells (A) and Mdr2−/−/p19ARF−/− MFB (B) were transfected with either an unsilencing siRNA (uns. siRNA) or a specific siRNA for NOX4 (NOX4 siRNA) as described in Figs. 4 and 5, respectively. In the case of HSC (A), cells were either not treated or treated with TGF-β (2 ng/ml), as indicated in the figure. Data shown correspond to the real-time PCR analysis of the mRNA levels of NOX4, NOX2 and NOX1, which were calculated relative to 18S expression. In order to compare the expression level among the different isoforms, we gave an arbitrary value of 1 to NOX4 expression under basal conditions in both HSC (A) and MFB (B), and all the data are referred to this value. Mean ± S.E.M. is shown and the specific value of the mean detailed above each bar. (TIF) [file pone.0045285.s007.tif]

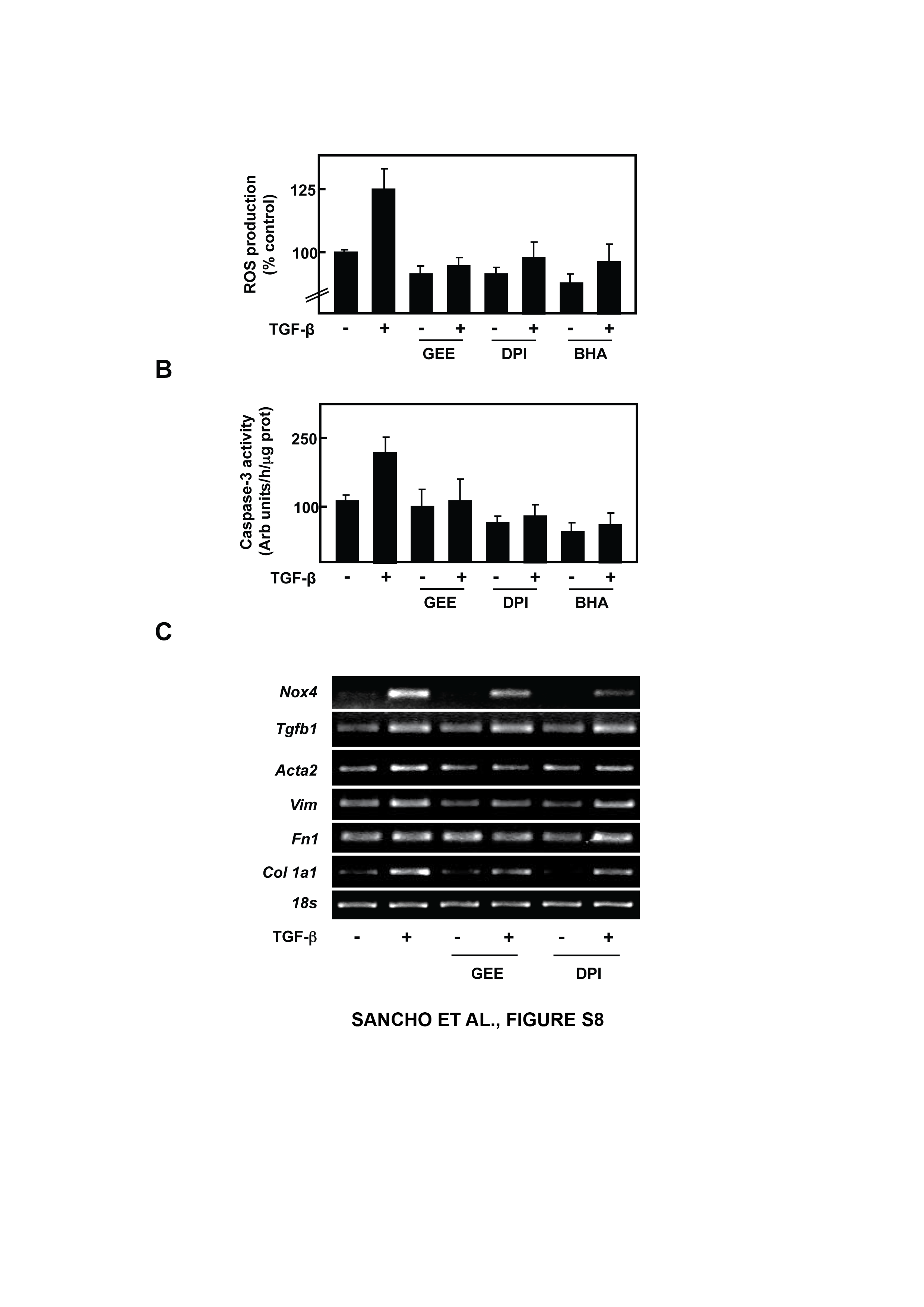

Supplement: Figure S8 — Role of antioxidants (GEE and BHA) or a NADPH oxidase inhibitor (DPI) on the effects of TGF-β on ROS production and apoptosis in hepatocytes (A and B, respectively) and activation of HSC (C). Cells were pre-incubated during 30 min with 1 μM DPI, 2 mM GEE or 200 μM BHA, as indicated in each figure, before adding 2 ng/mlof TGF-β. The same concentrations of the agents were maintained during all the TGF-β treatment. A. After 3 h of treatment in hepatocytes, intracellular content of ROS was analyzed through fluorimetric assay as detailed in the Materials and Methods section. Data represent the mean ± S.E.M. of 3 independent experiments in triplicate and are referred to the value in untreated cells (100%). B. After 16 h of treatment in hepatocytes, proteins were collected for caspase-3 analysis. Data represent the mean ± S.E.M. of 3 independent experiments in duplicate and are expressed as arbitrary units per hour and per microgram of protein. C. After 48 h of treatment in HSC, RNA was collected for analysis of expression by RT-PCR of the genes detailed in the figure. A representative experiment out of 3 is shown. (TIF) [file pone.0045285.s008.tif]
