# Supplementary material for: NADPH Oxidase NOX4 Mediates Stellate Cell Activation and Hepatocyte Cell Death during Liver Fibrosis Development
Source: PLoS One. 2012 Sep 26;7(9):e45285. doi: 10.1371/journal.pone.0045285 (PMC3458844; doi:10.1371/journal.pone.0045285)
Supplement: Table S1 — Mouse primer sequences used for semiquantitative PCR. (DOC) [file pone.0045285.s009.doc]

**Table S1. Mouse primer sequences used for semiquantitative PCR**

|  | **Forward** | **Reverse** |
| --- | --- | --- |
| **Col1a1** | TCAAGGTCTACTGCAACATGG | GCGAAAGCATTTGCCAAGAA |
| **Fn1** | GGTTTCCCATTACGCCATTG | ATTCTCCCTTTCCATTCCCG |
| **Nox4** | GGAAGCCCATTTGAGGAGTCAC | CTGAGGTACAGCTGGATGTTCA |
| **Acta2** | CCGAGATCTCACCGACTACC | TCCAGAGCGACATAGCACAG |
| **Vim** | TCCGCCAGCAGTATGAAAG | TGGGTGTCAACCAGAGGAAG |
| **Tgfb1** | GTGAAACGGAAGCGCATCGAAG | AGCCGGTTACCAAGGTAACGCC |
| **Cdh1** | CGTGATGAAGGTCTCAGCC | ATGGGGGCTTCATTCAC |
| **Snai1** | GCAGCTGGCCAGGCTCTCGGTGGC | GTAGCTGGGTCAGCGAGGGCCTCC |
| **18s** | GCGAAAGCATTTGCCAAGAA | CATCACAGACCTGTTATTGC |
