# Supplementary material for: NADPH Oxidase NOX4 Mediates Stellate Cell Activation and Hepatocyte Cell Death during Liver Fibrosis Development
Source: PLoS One. 2012 Sep 26;7(9):e45285. doi: 10.1371/journal.pone.0045285 (PMC3458844; doi:10.1371/journal.pone.0045285)
Supplement: Table S2 — Mouse and human primer sequences used for quantitative real-time PCR. (DOC) [file pone.0045285.s010.doc]

**Table S2. Mouse and human primer sequences used for quantitative real-time PCR**

|  |  | Forward | Reverse |
| --- | --- | --- | --- |
| **Mouse** | Nox1 | TCCTTCGCTTTTATCGCTCC | TCGCTTCCTCATCTGCAATTC |
|  | Nox2 | TCCTATGTTCCTGTACCTTTGTG | GTCCCACCTCCATCTTGAATC |
|  | Nox4 | TCCAAGCTCATTTCCCACAG | CGGAGTTCCATTACATCAGAGG |
|  | Acta2 | CCGAGATCTCACCGACTACC | AGG TGG TTT CGT GGA TGC |
|  | Vim | CATTGAGATCGCCACCTACAG | AGGAGTGTTCTTTTGGAGTGG |
|  | Col1a1 | TCAAGGTCTACTGCAACATGG | TGTAGGTGAAGCGACTGTTG |
|  | Desmin | CTAAAGGATGAGATGGCCCG | GAAGGTCTGGATAGGAAGGTT |
|  | Fn1 | GGTTTCCCATTACGCCATTG | ATTCTCCCTTTCCATTCCCG |
|  | Tgfb1 | CCTGAGTGGCTGTCTTTTGA | GTGGAGTACATTATCTTTGCTG |
|  | Tgfbr1 | CCAAACCACAGAGTAGGCAC | ACCAATAGAACAGCGTCGAG |
|  | 18s | CGAGACTCTGGCATGCTAA | CATCACAGACCTGTTATTGC |
| **Human** | TGFB1 | GGCAGCTGTACATTGACTTCC | CCTTGCTGTACTGCGTGTCC |
|  | TGFB2 | GAGTGCCTGAACAACGGATT | CATTCGCCTTCTGCTCTTG |
|  | TGFB3 | TCAAGAAGAAGAGGGTGGAA | CGGGTGCTGTTGTAAAGG |
|  | TGFBRI | AAGTCATCACCTGGCCTTGG | TCGATGGTGAATGACAGTGC |
|  | TGFBRII | GTGTGCCAACAACATCAACC | AGTGTTCTGCTTCAGCTTGG |
|  | NOX1 | CACAAGAAAAATCCTTGGGTCA | GACAGCAGATTGCGACACACA |
|  | NOX2 | CTGCATGCTGATTCTCTTGC | TTCCTGTCCAGTTGTCTTCG |
|  | NOX4 | CCTCAACTGCAGCCTTATCC | CAACAATCTCCTGGTTCTCC |
|  | HPRT1 | AGCCCTGGCGTCGTGATTAGT | CGAGCAAGACGTTCAGTCCTGT |
